# Supplementary material for: Improved detection of tumor suppressor events in single-cell RNA-Seq data
Source: NPJ Genom Med. 2020 Oct 7;5:43. doi: 10.1038/s41525-020-00151-y (PMC7541488; doi:10.1038/s41525-020-00151-y)
Supplement: Supplementary file 2 — Reporting Summary [file 41525_2020_151_MOESM2_ESM.pdf]

nature research

Corresponding author(s): Andrew E Teschendorff

Last updated by author(s): Aug 18, 2020

Reporting Summary

Nature Research wishes to improve the reproducibility of the work that we publish. This form provides structure for consistency and transparency in reporting. For further information on Nature Research policies, see Authors & Referees and the Editorial Policy Checklist.

Statistics

For all statistical analyses, confirm that the following items are present in the figure legend, table legend, main text, or Methods section.

n/a	Confirmed	
☐	☒	The exact sample size (*n*) for each experimental group/condition, given as a discrete number and unit of measurement
☐	☒	A statement on whether measurements were taken from distinct samples or whether the same sample was measured repeatedly
☐	☒	The statistical test(s) used AND whether they are one- or two-sided<br>*Only common tests should be described solely by name; describe more complex techniques in the Methods section.*
☐	☒	A description of all covariates tested
☐	☒	A description of any assumptions or corrections, such as tests of normality and adjustment for multiple comparisons
☐	☒	A full description of the statistical parameters including central tendency (e.g. means) or other basic estimates (e.g. regression coefficient) AND variation (e.g. standard deviation) or associated estimates of uncertainty (e.g. confidence intervals)
☐	☒	For null hypothesis testing, the test statistic (e.g. *F*, *t*, *r*) with confidence intervals, effect sizes, degrees of freedom and *P* value noted<br>*Give P values as exact values whenever suitable.*
☒	☐	For Bayesian analysis, information on the choice of priors and Markov chain Monte Carlo settings
☒	☐	For hierarchical and complex designs, identification of the appropriate level for tests and full reporting of outcomes
☐	☒	Estimates of effect sizes (e.g. Cohen's *d*, Pearson's *r*), indicating how they were calculated

Our web collection on statistics for biologists contains articles on many of the points above.

Software and code

Policy information about availability of computer code

Data collection	No software was used to collect data. All data was downloaded from public repositories.
Data analysis	The following main Bioconductor/R-packages were used: SCIRA_0.9.1, corpcor_1.6.9, limma_3.38.3, parallel_3.5.2, GENIE3_1.4.0, RcisTarget_1.2.0 and AUCell_1.4.1, OCplus_1.56.0, destiny_2.14.0 , viper_1.18.1, org.Hs.eg.db_3.8.2, sna_2.5, scales_1.1.1, network_1.16.0, ggplot2_3.3.1

For manuscripts utilizing custom algorithms or software that are central to the research but not yet described in published literature, software must be made available to editors/reviewers. We strongly encourage code deposition in a community repository (e.g. GitHub). See the Nature Research guidelines for submitting code & software for further information.

Data

Policy information about availability of data

All manuscripts must include a data availability statement. This statement should provide the following information, where applicable:

- Accession codes, unique identifiers, or web links for publicly available datasets
- A list of figures that have associated raw data
- A description of any restrictions on data availability

Data analyzed in this manuscript is already publicly available from the following GEO (www.ncbi.nlm.nih.gov/geo/) accession numbers: GSE52583, GSE90047, GSE115931, GSE118184, GSE81861, GSE131391, and from ArrayExpress (www.ebi.ac.uk/arrayexpress) under accession number E-MTAB-6149.

Field-specific reporting

Please select the one below that is the best fit for your research. If you are not sure, read the appropriate sections before making your selection.

☒ Life sciences ☐ Behavioural & social sciences ☐ Ecological, evolutionary & environmental sciences

For a reference copy of the document with all sections, see nature.com/documents/nr-reporting-summary-flat.pdf

Life sciences study design

All studies must disclose on these points even when the disclosure is negative.

Sample size	Yes, a key component of SCIRA is a power calculation to show that with the GTEX RNA-Seq bulk dataset we are sufficiently powered to detect transcription factors xpressed in only 5% of cells in a given tissue. We provide full details of the formula used to estimate power.
Data exclusions	No specific exclusions, other than the single-cells not passing standard QC criteria, as described in the respective publications were the scRNA-Seq data were first published.
Replication	All computations performed here are reproducible, i.e. don't require setting of a seed variable. As far as Monte-Carlo randomization analysis is concerned, we verified that the number of Monte-Carlo runs was sufficient to guarantee reproducibility.
Randomization	Not applicable to this study
Blinding	Not applicable to this study

Reporting for specific materials, systems and methods

We require information from authors about some types of materials, experimental systems and methods used in many studies. Here, indicate whether each material, system or method listed is relevant to your study. If you are not sure if a list item applies to your research, read the appropriate section before selecting a response.

Materials & experimental systems

n/a	Involved in the study	
☒	☐	Antibodies
☒	☐	Eukaryotic cell lines
☒	☐	Palaeontology
☒	☐	Animals and other organisms
☒	☐	Human research participants
☒	☐	Clinical data

Methods

n/a	Involved in the study	
☒	☐	ChIP-seq
☒	☐	Flow cytometry
☒	☐	MRI-based neuroimaging
